# Supplementary figures and images for: Development of Pomegranate Peel Extract and Nano ZnO Co-Reinforced Polylactic Acid Film for Active Food Packaging
Source: Membranes (Basel). 2022 Nov 6;12(11):1108. doi: 10.3390/membranes12111108 (PMC9694470; doi:10.3390/membranes12111108)

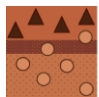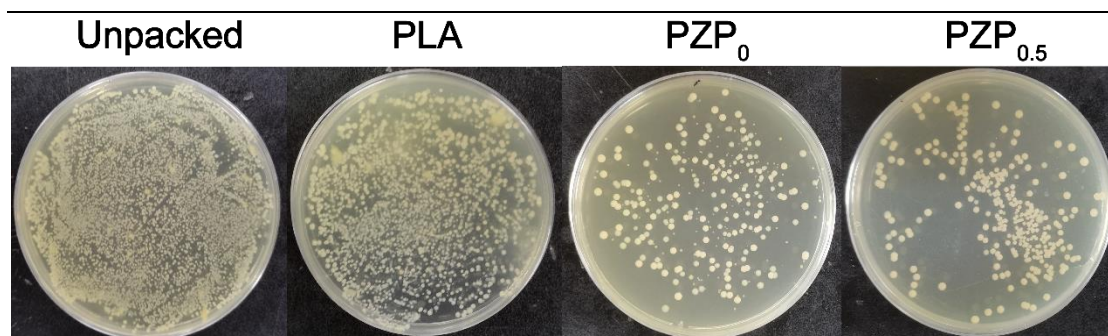

**Figure S1.** Plate count results of 15-day cherry tomatoes from four groups

Supplement: Supplementary file 1 [file membranes-12-01108-s001.zip › membranes-1961191-supplementary.pdf]
